# 341. GeneXpert Ultra for the Diagnosis of Extrapulmonary Lymphnode Tuberculosis – A Diagnostic Study from High Tuberculosis Burden Country

**DOI:** 10.1093/ofid/ofac492.419

**Published:** 2022-12-15

**Authors:** Santhanam Naguthevar, Deepak Kumar, Akshatha Ravindra, Subhashree Samantaray, Sarika P Kombade, Gopal Krishna Bohra, Durga Shankar Meena, Naresh Kumar Midha, Vidhi Jain, M K Garg, Kuldeep Singh

**Affiliations:** AIIMS JODHPUR, Jodhpur, Rajasthan, India; AIIMS jodhpur, Jodhpur, Rajasthan, India; AIIMS Jodhpur, Jodhpur, Rajasthan, India; AIIMS JODHPUR, Jodhpur, Rajasthan, India; AIIMS, Jodhpur, Rajasthan, India; AIIMS, Jodhpur, Rajasthan, India; AIIMS, Jodhpur, Rajasthan, India; AIIMS, Jodhpur, Rajasthan, India; AIIMS, Jodhpur, Rajasthan, India; AIIMS, Jodhpur, Rajasthan, India; AIIMS, Jodhpur, Rajasthan, India

## Abstract

**Background:**

Extrapulmonary tuberculosis (EPTB) is quite prevalent in India, ranging from 8.3% and 13.1%. Diagnosis of EPTB is often challenging as it resembles other granulomatous conditions pathologically. TB culture remains the gold standard for definitive diagnosis, but it takes longer time and WHO has recommended the use of GeneXpert Ultra for rapid diagnosis. This study aims the utility of GeneXpert Ultra for diagnosis of tubercular adenitis.

**Methods:**

This prospective study included the suspected tubercular adenitis patients with lymphnode size of ≥ 20mm. Patients diagnosed with other aetiology of adenitis were excluded. Excision biopsy was performed in all patients. Tissue samples were sent for Ziehl Neelson (ZN) stain, GeneXpert Ultra, histopathology and mycobacterial culture (BACTEC). The sensitivity, specificity, positive predictive value (PPV), and Negative predictive value (NPV) of GeneXpert Ultra were analysed in comparison with culture results.

**Results:**

A total of 85 patients Lymph node were included, of which 60% (n=51) had cervical LN, 15%(n=13) abdominal LN, 12%(n=11) mediastinal LN, and 15%(n=11) others. Most of the patients (97%) HPE findings of granulomatous inflammation of which only 20%(n=17) were ZN stain Positive. TB culture showed positivity of 56.47%(n=48) . *Mycobacterial tuberculosis* was detected in 69%(n=59) of patients by GeneXpert Ultra & none of them showed Rifampicin resistance. The Sensitivity, specificity, PPV & NPV for GeneXpert Ultra were found 85.4%, 51.3%, 25.06% and 94.8%, respectively.

**Conclusion:**

GeneXpert Ultra showed excellent sensitivity and negative predictive value for the diagnosis of TB Lymphadenitis. Further this method detects Rifampin sensitivity which is crucial for initiation of appropriate antitubercular drugs.

**Disclosures:**

**All Authors**: No reported disclosures.

